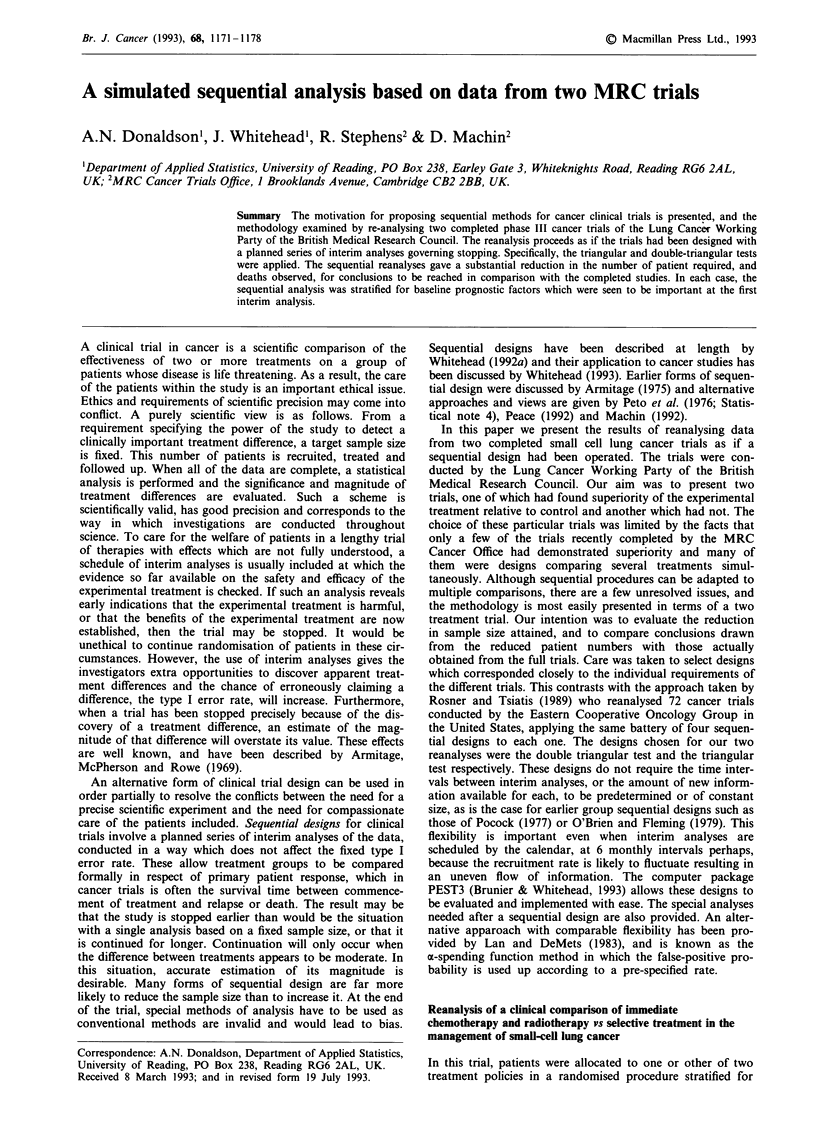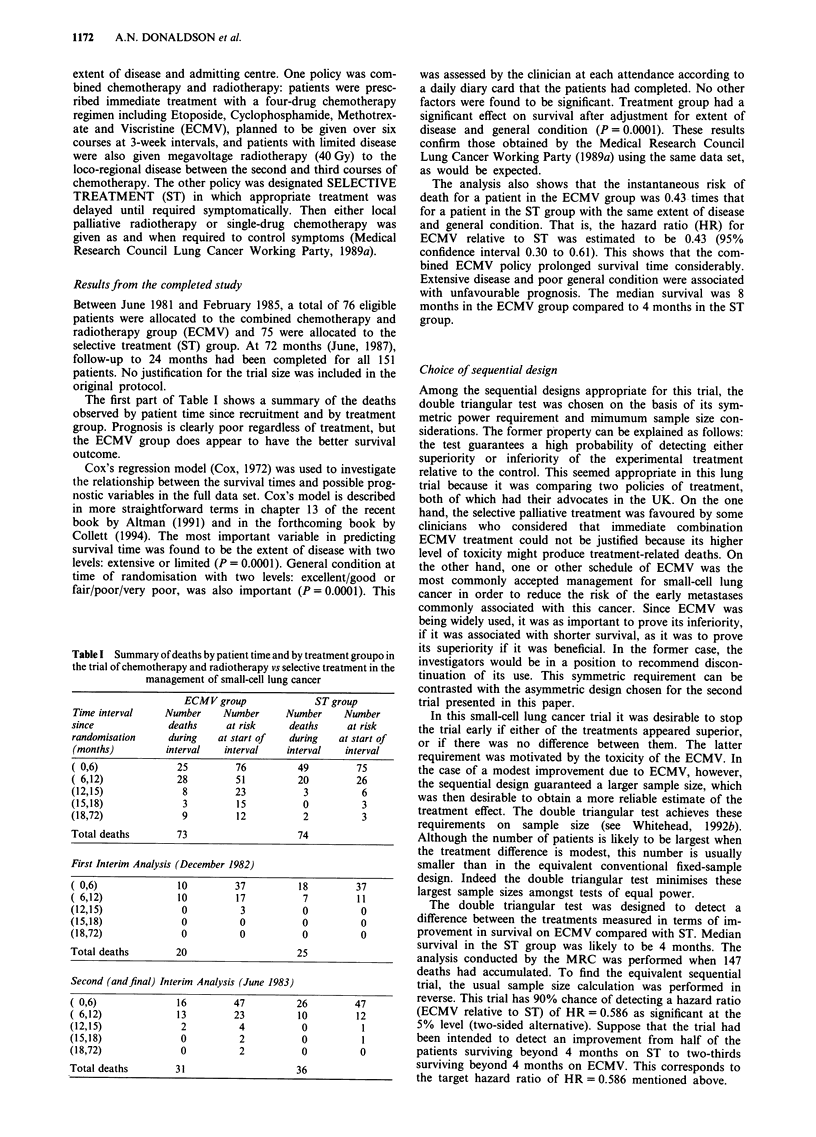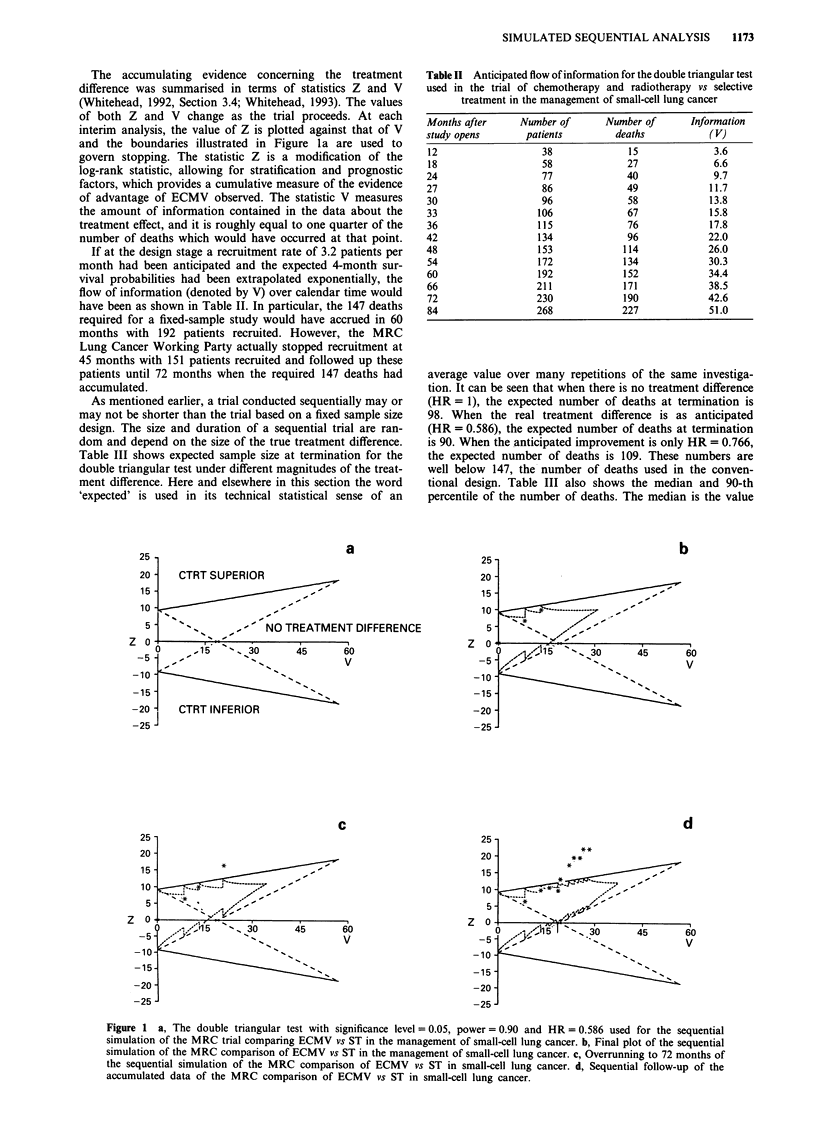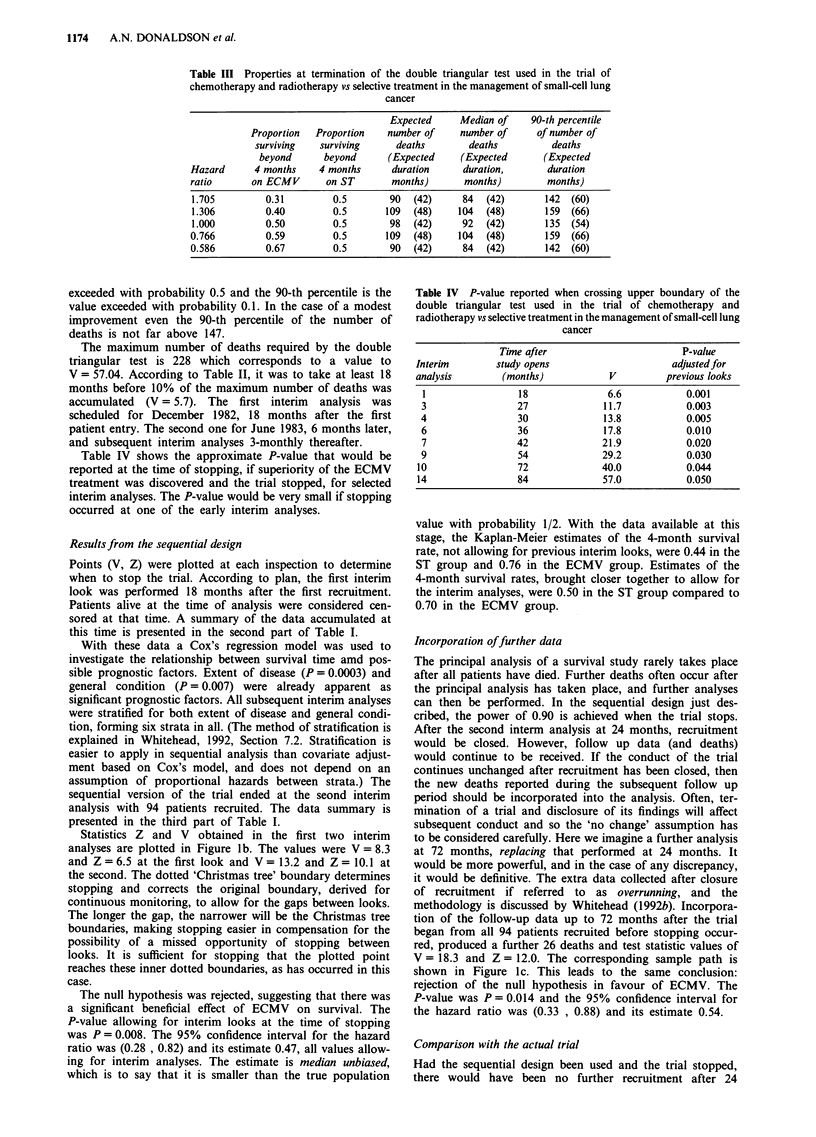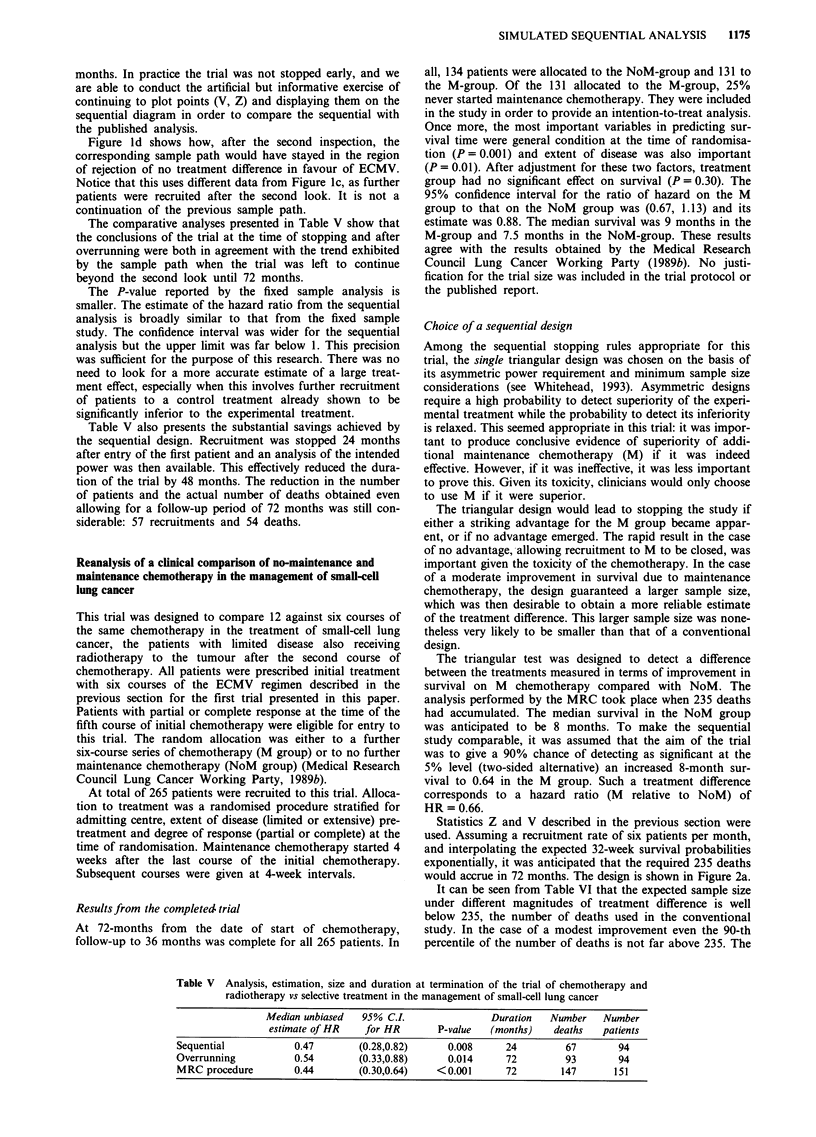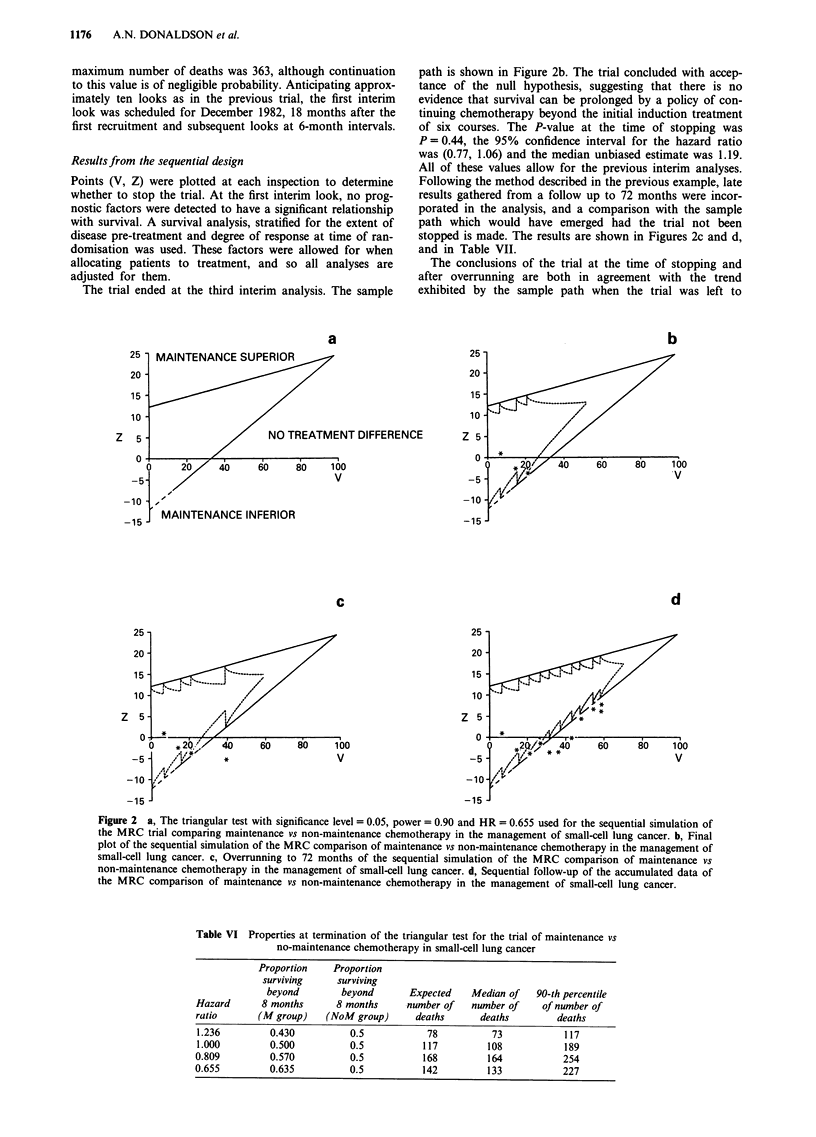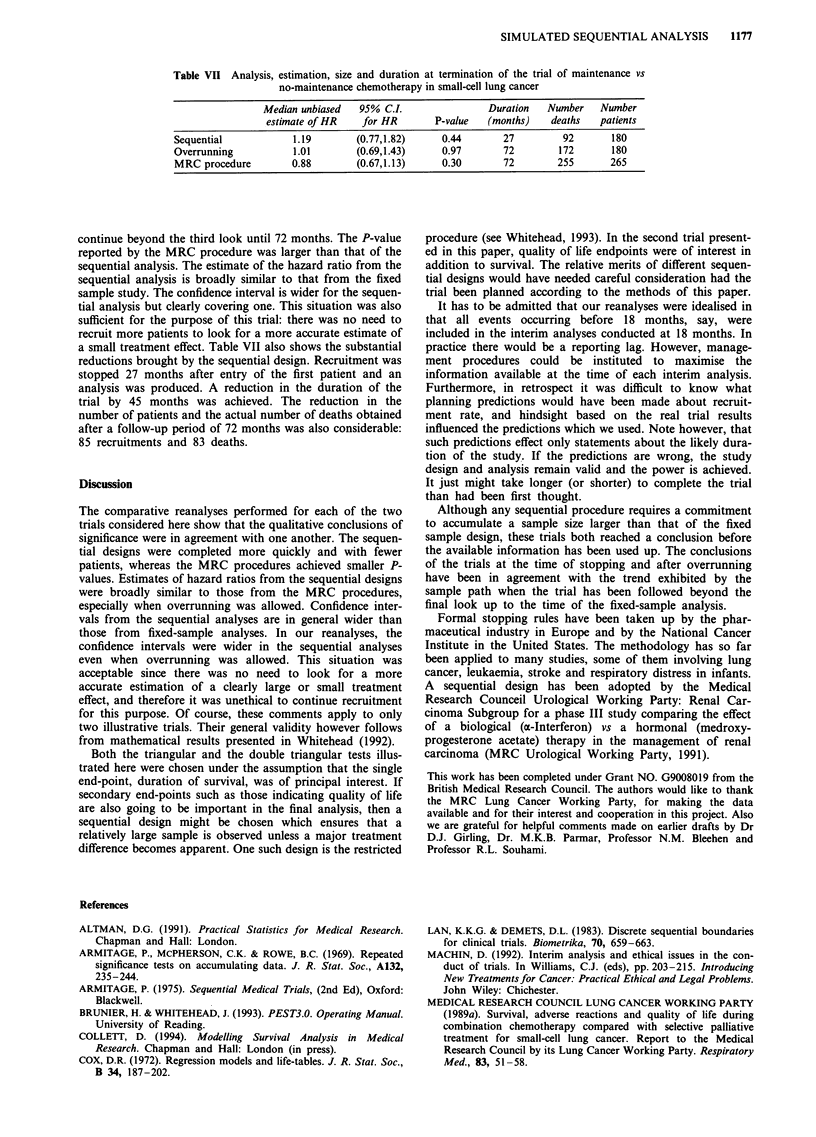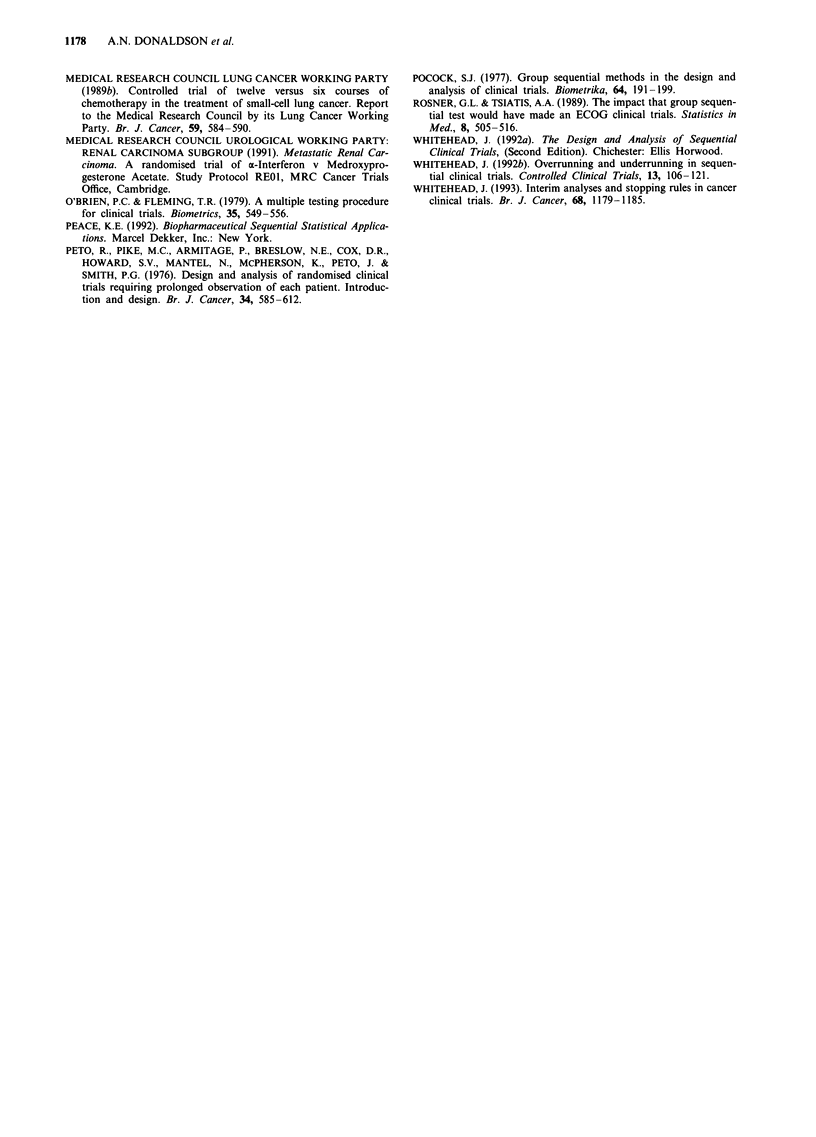# A simulated sequential analysis based on data from two MRC trials.

**DOI:** 10.1038/bjc.1993.499

**Published:** 1993-12

**Authors:** A. N. Donaldson, J. Whitehead, R. Stephens, D. Machin

**Affiliations:** Department of Applied Statistics, University of Reading, UK.

## Abstract

The motivation for proposing sequential methods for cancer clinical trials is presented, and the methodology examined by re-analysing two completed phase III cancer trials of the Lung Cancer Working Party of the British Medical Research Council. The reanalysis proceeds as if the trials had been designed with a planned series of interim analyses governing stopping. Specifically, the triangular and double-triangular tests were applied. The sequential reanalysis gave a substantial reduction in the number of patient required, and deaths observed, for conclusions to be reached in comparison with the completed studies. In each case, the sequential analysis was stratified for baseline prognostic factors which were seen to be important at the first interim analysis.


Br. J. Cancer (1993), 68, 1171–1178 © Macmillan Press Ltd., 1993

### A simulated sequential analysis based on data from two MRC trials

A.N. Donaldson[1], J. Whitehead[1], R. Stephens[2] & D. Machin[2]

[1]*Department of Applied Statistics, University of Reading, PO Box 238, Earley Gate 3, Whiteknights Road, Reading RG6 2AL, UK;* [2]*MRC Cancer Trials Office, 1 Brooklands Avenue, Cambridge CB2 2BB, UK.*

**Summary** The motivation for proposing sequential methods for cancer clinical trials is presented, and the methodology examined by re-analysing two completed phase III cancer trials of the Lung Cancer Working Party of the British Medical Research Council. The reanalysis proceeds as if the trials had been designed with a planned series of interim analyses governing stopping. Specifically, the triangular and double-triangular tests were applied. The sequential reanalyses gave a substantial reduction in the number of patient required, and deaths observed, for conclusions to be reached in comparison with the completed studies. In each case, the sequential analysis was stratified for baseline prognostic factors which were seen to be important at the first interim analysis.

A clinical trial in cancer is a scientific comparison of the effectiveness of two or more treatments on a group of patients whose disease is life threatening. As a result, the care of the patients within the study is an important ethical issue. Ethics and requirements of scientific precision may come into conflict. A purely scientific view is as follows. From a requirement specifying the power of the study to detect a clinically important treatment difference, a target sample size is fixed. This number of patients is recruited, treated and followed up. When all of the data are complete, a statistical analysis is performed and the significance and magnitude of treatment differences are evaluated. Such a scheme is scientifically valid, has good precision and corresponds to the way in which investigations are conducted throughout science. To care for the welfare of patients in a lengthy trial of therapies with effects which are not fully understood, a schedule of interim analyses is usually included at which the evidence so far available on the safety and efficacy of the experimental treatment is checked. If such an analysis reveals early indications that the experimental treatment is harmful, or that the benefits of the experimental treatment are now established, then the trial may be stopped. It would be unethical to continue randomisation of patients in these circumstances. However, the use of interim analyses gives the investigators extra opportunities to discover apparent treatment differences and the chance of erroneously claiming a difference, the type I error rate, will increase. Furthermore, when a trial has been stopped precisely because of the discovery of a treatment difference, an estimate of the magnitude of that difference will overstate its value. These effects are well known, and have been described by Armitage, McPherson and Rowe (1969).

An alternative form of clinical trial design can be used in order partially to resolve the conflicts between the need for a precise scientific experiment and the need for compassionate care of the patients included. *Sequential designs* for clinical trials involve a planned series of interim analyses of the data, conducted in a way which does not affect the fixed type I error rate. These allow treatment groups to be compared formally in respect of primary patient response, which in cancer trials is often the survival time between commencement of treatment and relapse or death. The result may be that the study is stopped earlier than would be the situation with a single analysis based on a fixed sample size, or that it is continued for longer. Continuation will only occur when the difference between treatments appears to be moderate. In this situation, accurate estimation of its magnitude is desirable. Many forms of sequential design are far more likely to reduce the sample size than to increase it. At the end of the trial, special methods of analysis have to be used as conventional methods are invalid and would lead to bias. Sequential designs have been described at length by Whitehead (1992*a*) and their application to cancer studies has been discussed by Whitehead (1993). Earlier forms of sequential design were discussed by Armitage (1975) and alternative approaches and views are given by Peto *et al.* (1976; Statistical note 4), Peace (1992) and Machin (1992).

In this paper we present the results of reanalysing data from two completed small cell lung cancer trials as if a sequential design had been operated. The trials were conducted by the Lung Cancer Working Party of the British Medical Research Council. Our aim was to present two trials, one of which had found superiority of the experimental treatment relative to control and another which had not. The choice of these particular trials was limited by the facts that only a few of the trials recently completed by the MRC Cancer Office had demonstrated superiority and many of them were designs comparing several treatments simultaneously. Although sequential procedures can be adapted to multiple comparisons, there are a few unresolved issues, and the methodology is most easily presented in terms of a two treatment trial. Our intention was to evaluate the reduction in sample size attained, and to compare conclusions drawn from the reduced patient numbers with those actually obtained from the full trials. Care was taken to select designs which corresponded closely to the individual requirements of the different trials. This contrasts with the approach taken by Rosner and Tsiatis (1989) who reanalysed 72 cancer trials conducted by the Eastern Cooperative Oncology Group in the United States, applying the same battery of four sequential designs to each one. The designs chosen for our two reanalyses were the double triangular test and the triangular test respectively. These designs do not require the time intervals between interim analyses, or the amount of new information available for each, to be predetermined or of constant size, as is the case for earlier group sequential designs such as those of Pocock (1977) or O'Brien and Fleming (1979). This flexibility is important even when interim analyses are scheduled by the calendar, at 6 monthly intervals perhaps, because the recruitment rate is likely to fluctuate resulting in an uneven flow of information. The computer package PEST3 (Brunier & Whitehead, 1993) allows these designs to be evaluated and implemented with ease. The special analyses needed after a sequential design are also provided. An alternative apparaoach with comparable flexibility has been provided by Lan and DeMets (1983), and is known as the α-spending function method in which the false-positive probability is used up according to a pre-specified rate.

### Reanalysis of a clinical comparison of immediate chemotherapy and radiotherapy *vs* selective treatment in the management of small-cell lung cancer

In this trial, patients were allocated to one or other of two treatment policies in a randomised procedure stratified for

Correspondence: A.N. Donaldson, Department of Applied Statistics, University of Reading, PO Box 238, Reading RG6 2AL, UK.
Received 8 March 1993; and in revised form 19 July 1993.

extent of disease and admitting centre. One policy was combined chemotherapy and radiotherapy: patients were prescribed immediate treatment with a four-drug chemotherapy regimen including Etoposide, Cyclophosphamide, Methotrexate and Viscristine (ECMV), planned to be given over six courses at 3-week intervals, and patients with limited disease were also given megavoltage radiotherapy (40 Gy) to the loco-regional disease between the second and third courses of chemotherapy. The other policy was designated SELECTIVE TREATMENT (ST) in which appropriate treatment was delayed until required symptomatically. Then either local palliative radiotherapy or single-drug chemotherapy was given as and when required to control symptoms (Medical Research Council Lung Cancer Working Party, 1989*a*).

*Results from the completed study*

Between June 1981 and February 1985, a total of 76 eligible patients were allocated to the combined chemotherapy and radiotherapy group (ECMV) and 75 were allocated to the selective treatment (ST) group. At 72 months (June, 1987), follow-up to 24 months had been completed for all 151 patients. No justification for the trial size was included in the original protocol.

The first part of Table I shows a summary of the deaths observed by patient time since recruitment and by treatment group. Prognosis is clearly poor regardless of treatment, but the ECMV group does appear to have the better survival outcome.

Cox's regression model (Cox, 1972) was used to investigate the relationship between the survival times and possible prognostic variables in the full data set. Cox's model is described in more straightforward terms in chapter 13 of the recent book by Altman (1991) and in the forthcoming book by Collett (1994). The most important variable in predicting survival time was found to be the extent of disease with two levels: extensive or limited ($P = 0.0001$). General condition at time of randomisation with two levels: excellent/good or fair/poor/very poor, was also important ($P = 0.0001$). This was assessed by the clinician at each attendance according to a daily diary card that the patients had completed. No other factors were found to be significant. Treatment group had a significant effect on survival after adjustment for extent of disease and general condition ($P = 0.0001$). These results confirm those obtained by the Medical Research Council Lung Cancer Working Party (1989*a*) using the same data set, as would be expected.

The analysis also shows that the instantaneous risk of death for a patient in the ECMV group was 0.43 times that for a patient in the ST group with the same extent of disease and general condition. That is, the hazard ratio (HR) for ECMV relative to ST was estimated to be 0.43 (95% confidence interval 0.30 to 0.61). This shows that the combined ECMV policy prolonged survival time considerably. Extensive disease and poor general condition were associated with unfavourable prognosis. The median survival was 8 months in the ECMV group compared to 4 months in the ST group.

**Table I** Summary of deaths by patient time and by treatment groupo in the trial of chemotherapy and radiotherapy *vs* selective treatment in the management of small-cell lung cancer

| | *ECMV group* | | *ST group* | |
|---|---|---|---|---|
| *Time interval since randomisation (months)* | *Number deaths during interval* | *Number at risk at start of interval* | *Number deaths during interval* | *Number at risk at start of interval* |
| ( 0,6) | 25 | 76 | 49 | 75 |
| ( 6,12) | 28 | 51 | 20 | 26 |
| (12,15) | 8 | 23 | 3 | 6 |
| (15,18) | 3 | 15 | 0 | 3 |
| (18,72) | 9 | 12 | 2 | 3 |
| Total deaths | 73 | | 74 | |
| *First Interim Analysis (December 1982)* | | | | |
| ( 0,6) | 10 | 37 | 18 | 37 |
| ( 6,12) | 10 | 17 | 7 | 11 |
| (12,15) | 0 | 3 | 0 | 0 |
| (15,18) | 0 | 0 | 0 | 0 |
| (18,72) | 0 | 0 | 0 | 0 |
| Total deaths | 20 | | 25 | |
| *Second (and final) Interim Analysis (June 1983)* | | | | |
| ( 0,6) | 16 | 47 | 26 | 47 |
| ( 6,12) | 13 | 23 | 10 | 12 |
| (12,15) | 2 | 4 | 0 | 1 |
| (15,18) | 0 | 2 | 0 | 1 |
| (18,72) | 0 | 2 | 0 | 0 |
| Total deaths | 31 | | 36 | |

*Choice of sequential design*

Among the sequential designs appropriate for this trial, the double triangular test was chosen on the basis of its symmetric power requirement and mimumum sample size considerations. The former property can be explained as follows: the test guarantees a high probability of detecting either superiority or inferiority of the experimental treatment relative to the control. This seemed appropriate in this lung trial because it was comparing two policies of treatment, both of which had their advocates in the UK. On the one hand, the selective palliative treatment was favoured by some clinicians who considered that immediate combination ECMV treatment could not be justified because its higher level of toxicity might produce treatment-related deaths. On the other hand, one or other schedule of ECMV was the most commonly accepted management for small-cell lung cancer in order to reduce the risk of the early metastases commonly associated with this cancer. Since ECMV was being widely used, it was as important to prove its inferiority, if it was associated with shorter survival, as it was to prove its superiority if it was beneficial. In the former case, the investigators would be in a position to recommend discontinuation of its use. This symmetric requirement can be contrasted with the asymmetric design chosen for the second trial presented in this paper.

In this small-cell lung cancer trial it was desirable to stop the trial early if either of the treatments appeared superior, or if there was no difference between them. The latter requirement was motivated by the toxicity of the ECMV. In the case of a modest improvement due to ECMV, however, the sequential design guaranteed a larger sample size, which was then desirable to obtain a more reliable estimate of the treatment effect. The double triangular test achieves these requirements on sample size (see Whitehead, 1992*b*). Although the number of patients is likely to be largest when the treatment difference is modest, this number is usually smaller than in the equivalent conventional fixed-sample design. Indeed the double triangular test minimises these largest sample sizes amongst tests of equal power.

The double triangular test was designed to detect a difference between the treatments measured in terms of improvement in survival on ECMV compared with ST. Median survival in the ST group was likely to be 4 months. The analysis conducted by the MRC was performed when 147 deaths had accumulated. To find the equivalent sequential trial, the usual sample size calculation was performed in reverse. This trial has 90% chance of detecting a hazard ratio (ECMV relative to ST) of $HR = 0.586$ as significant at the 5% level (two-sided alternative). Suppose that the trial had been intended to detect an improvement from half of the patients surviving beyond 4 months on ST to two-thirds surviving beyond 4 months on ECMV. This corresponds to the target hazard ratio of $HR = 0.586$ mentioned above.

The accumulating evidence concerning the treatment difference was summarised in terms of statistics Z and V (Whitehead, 1992, Section 3.4; Whitehead, 1993). The values of both Z and V change as the trial proceeds. At each interim analysis, the value of Z is plotted against that of V and the boundaries illustrated in Figure 1a are used to govern stopping. The statistic Z is a modification of the log-rank statistic, allowing for stratification and prognostic factors, which provides a cumulative measure of the evidence of advantage of ECMV observed. The statistic V measures the amount of information contained in the data about the treatment effect, and it is roughly equal to one quarter of the number of deaths which would have occurred at that point.

If at the design stage a recruitment rate of 3.2 patients per month had been anticipated and the expected 4-month survival probabilities had been extrapolated exponentially, the flow of information (denoted by V) over calendar time would have been as shown in Table II. In particular, the 147 deaths required for a fixed-sample study would have accrued in 60 months with 192 patients recruited. However, the MRC Lung Cancer Working Party actually stopped recruitment at 45 months with 151 patients recruited and followed up these patients until 72 months when the required 147 deaths had accumulated.

As mentioned earlier, a trial conducted sequentially may or may not be shorter than the trial based on a fixed sample size design. The size and duration of a sequential trial are random and depend on the size of the true treatment difference. Table III shows expected sample size at termination for the double triangular test under different magnitudes of the treatment difference. Here and elsewhere in this section the word 'expected' is used in its technical statistical sense of an

**Table II** Anticipated flow of information for the double triangular test used in the trial of chemotherapy and radiotherapy *vs* selective treatment in the management of small-cell lung cancer

| *Months after study opens* | *Number of patients* | *Number of deaths* | *Information (V)* |
|---|---|---|---|
| 12 | 38 | 15 | 3.6 |
| 18 | 58 | 27 | 6.6 |
| 24 | 77 | 40 | 9.7 |
| 27 | 86 | 49 | 11.7 |
| 30 | 96 | 58 | 13.8 |
| 33 | 106 | 67 | 15.8 |
| 36 | 115 | 76 | 17.8 |
| 42 | 134 | 96 | 22.0 |
| 48 | 153 | 114 | 26.0 |
| 54 | 172 | 134 | 30.3 |
| 60 | 192 | 152 | 34.4 |
| 66 | 211 | 171 | 38.5 |
| 72 | 230 | 190 | 42.6 |
| 84 | 268 | 227 | 51.0 |

average value over many repetitions of the same investigation. It can be seen that when there is no treatment difference (HR = 1), the expected number of deaths at termination is 98. When the real treatment difference is as anticipated (HR = 0.586), the expected number of deaths at termination is 90. When the anticipated improvement is only HR = 0.766, the expected number of deaths is 109. These numbers are well below 147, the number of deaths used in the conventional design. Table III also shows the median and 90-th percentile of the number of deaths. The median is the value

**Figure 1** **a**, The double triangular test with significance level = 0.05, power = 0.90 and HR = 0.586 used for the sequential simulation of the MRC trial comparing ECMV *vs* ST in the management of small-cell lung cancer. **b**, Final plot of the sequential simulation of the MRC comparison of ECMV *vs* ST in the management of small-cell lung cancer. **c**, Overrunning to 72 months of the sequential simulation of the MRC comparison of ECMV *vs* ST in small-cell lung cancer. **d**, Sequential follow-up of the accumulated data of the MRC comparison of ECMV *vs* ST in small-cell lung cancer.

**Table III** Properties at termination of the double triangular test used in the trial of chemotherapy and radiotherapy *vs* selective treatment in the management of small-cell lung cancer

| *Hazard ratio* | *Proportion surviving beyond 4 months on ECMV* | *Proportion surviving beyond 4 months on ST* | *Expected number of deaths (Expected duration months)* | *Median of number of deaths (Expected duration, months)* | *90-th percentile of number of deaths (Expected duration months)* |
|---|---|---|---|---|---|
| 1.705 | 0.31 | 0.5 | 90 (42) | 84 (42) | 142 (60) |
| 1.306 | 0.40 | 0.5 | 109 (48) | 104 (48) | 159 (66) |
| 1.000 | 0.50 | 0.5 | 98 (42) | 92 (42) | 135 (54) |
| 0.766 | 0.59 | 0.5 | 109 (48) | 104 (48) | 159 (66) |
| 0.586 | 0.67 | 0.5 | 90 (42) | 84 (42) | 142 (60) |

exceeded with probability 0.5 and the 90-th percentile is the value exceeded with probability 0.1. In the case of a modest improvement even the 90-th percentile of the number of deaths is not far above 147.

The maximum number of deaths required by the double triangular test is 228 which corresponds to a value to V = 57.04. According to Table II, it was to take at least 18 months before 10% of the maximum number of deaths was accumulated (V = 5.7). The first interim analysis was scheduled for December 1982, 18 months after the first patient entry. The second one for June 1983, 6 months later, and subsequent interim analyses 3-monthly thereafter.

Table IV shows the approximate *P*-value that would be reported at the time of stopping, if superiority of the ECMV treatment was discovered and the trial stopped, for selected interim analyses. The *P*-value would be very small if stopping occurred at one of the early interim analyses.

**Table IV** *P*-value reported when crossing upper boundary of the double triangular test used in the trial of chemotherapy and radiotherapy *vs* selective treatment in the management of small-cell lung cancer

| *Interim analysis* | *Time after study opens (months)* | *V* | P-*value adjusted for previous looks* |
|---|---|---|---|
| 1 | 18 | 6.6 | 0.001 |
| 3 | 27 | 11.7 | 0.003 |
| 4 | 30 | 13.8 | 0.005 |
| 6 | 36 | 17.8 | 0.010 |
| 7 | 42 | 21.9 | 0.020 |
| 9 | 54 | 29.2 | 0.030 |
| 10 | 72 | 40.0 | 0.044 |
| 14 | 84 | 57.0 | 0.050 |

*Results from the sequential design*

Points (V, Z) were plotted at each inspection to determine when to stop the trial. According to plan, the first interim look was performed 18 months after the first recruitment. Patients alive at the time of analysis were considered censored at that time. A summary of the data accumulated at this time is presented in the second part of Table I.

With these data a Cox's regression model was used to investigate the relationship between survival time amd possible prognostic factors. Extent of disease ($P = 0.0003$) and general condition ($P = 0.007$) were already apparent as significant prognostic factors. All subsequent interim analyses were stratified for both extent of disease and general condition, forming six strata in all. (The method of stratification is explained in Whitehead, 1992, Section 7.2. Stratification is easier to apply in sequential analysis than covariate adjustment based on Cox's model, and does not depend on an assumption of proportional hazards between strata.) The sequential version of the trial ended at the seond interim analysis with 94 patients recruited. The data summary is presented in the third part of Table I.

Statistics Z and V obtained in the first two interim analyses are plotted in Figure 1b. The values were V = 8.3 and Z = 6.5 at the first look and V = 13.2 and Z = 10.1 at the second. The dotted 'Christmas tree' boundary determines stopping and corrects the original boundary, derived for continuous monitoring, to allow for the gaps between looks. The longer the gap, the narrower will be the Christmas tree boundaries, making stopping easier in compensation for the possibility of a missed opportunity of stopping between looks. It is sufficient for stopping that the plotted point reaches these inner dotted boundaries, as has occurred in this case.

The null hypothesis was rejected, suggesting that there was a significant beneficial effect of ECMV on survival. The *P*-value allowing for interim looks at the time of stopping was $P = 0.008$. The 95% confidence interval for the hazard ratio was (0.28 , 0.82) and its estimate 0.47, all values allowing for interim analyses. The estimate is *median unbiased*, which is to say that it is smaller than the true population value with probability 1/2. With the data available at this stage, the Kaplan-Meier estimates of the 4-month survival rate, not allowing for previous interim looks, were 0.44 in the ST group and 0.76 in the ECMV group. Estimates of the 4-month survival rates, brought closer together to allow for the interim analyses, were 0.50 in the ST group compared to 0.70 in the ECMV group.

*Incorporation of further data*

The principal analysis of a survival study rarely takes place after all patients have died. Further deaths often occur after the principal analysis has taken place, and further analyses can then be performed. In the sequential design just described, the power of 0.90 is achieved when the trial stops. After the second interm analysis at 24 months, recruitment would be closed. However, follow up data (and deaths) would continue to be received. If the conduct of the trial continues unchanged after recruitment has been closed, then the new deaths reported during the subsequent follow up period should be incorporated into the analysis. Often, termination of a trial and disclosure of its findings will affect subsequent conduct and so the 'no change' assumption has to be considered carefully. Here we imagine a further analysis at 72 months, *replacing* that performed at 24 months. It would be more powerful, and in the case of any discrepancy, it would be definitive. The extra data collected after closure of recruitment if referred to as *overrunning*, and the methodology is discussed by Whitehead (1992*b*). Incorporation of the follow-up data up to 72 months after the trial began from all 94 patients recruited before stopping occurred, produced a further 26 deaths and test statistic values of V = 18.3 and Z = 12.0. The corresponding sample path is shown in Figure 1c. This leads to the same conclusion: rejection of the null hypothesis in favour of ECMV. The *P*-value was $P = 0.014$ and the 95% confidence interval for the hazard ratio was (0.33 , 0.88) and its estimate 0.54.

*Comparison with the actual trial*

Had the sequential design been used and the trial stopped, there would have been no further recruitment after 24

months. In practice the trial was not stopped early, and we are able to conduct the artificial but informative exercise of continuing to plot points (V, Z) and displaying them on the sequential diagram in order to compare the sequential with the published analysis.

Figure 1d shows how, after the second inspection, the corresponding sample path would have stayed in the region of rejection of no treatment difference in favour of ECMV. Notice that this uses different data from Figure 1c, as further patients were recruited after the second look. It is not a continuation of the previous sample path.

The comparative analyses presented in Table V show that the conclusions of the trial at the time of stopping and after overrunning were both in agreement with the trend exhibited by the sample path when the trial was left to continue beyond the second look until 72 months.

The *P*-value reported by the fixed sample analysis is smaller. The estimate of the hazard ratio from the sequential analysis is broadly similar to that from the fixed sample study. The confidence interval was wider for the sequential analysis but the upper limit was far below 1. This precision was sufficient for the purpose of this research. There was no need to look for a more accurate estimate of a large treatment effect, especially when this involves further recruitment of patients to a control treatment already shown to be significantly inferior to the experimental treatment.

Table V also presents the substantial savings achieved by the sequential design. Recruitment was stopped 24 months after entry of the first patient and an analysis of the intended power was then available. This effectively reduced the duration of the trial by 48 months. The reduction in the number of patients and the actual number of deaths obtained even allowing for a follow-up period of 72 months was still considerable: 57 recruitments and 54 deaths.

### Reanalysis of a clinical comparison of no-maintenance and maintenance chemotherapy in the management of small-cell lung cancer

This trial was designed to compare 12 against six courses of the same chemotherapy in the treatment of small-cell lung cancer, the patients with limited disease also receiving radiotherapy to the tumour after the second course of chemotherapy. All patients were prescribed initial treatment with six courses of the ECMV regimen described in the previous section for the first trial presented in this paper. Patients with partial or complete response at the time of the fifth course of initial chemotherapy were eligible for entry to this trial. The random allocation was either to a further six-course series of chemotherapy (M group) or to no further maintenance chemotherapy (NoM group) (Medical Research Council Lung Cancer Working Party, 1989*b*).

At total of 265 patients were recruited to this trial. Allocation to treatment was a randomised procedure stratified for admitting centre, extent of disease (limited or extensive) pretreatment and degree of response (partial or complete) at the time of randomisation. Maintenance chemotherapy started 4 weeks after the last course of the initial chemotherapy. Subsequent courses were given at 4-week intervals.

### *Results from the completed trial*

At 72-months from the date of start of chemotherapy, follow-up to 36 months was complete for all 265 patients. In all, 134 patients were allocated to the NoM-group and 131 to the M-group. Of the 131 allocated to the M-group, 25% never started maintenance chemotherapy. They were included in the study in order to provide an intention-to-treat analysis. Once more, the most important variables in predicting survival time were general condition at the time of randomisation ($P = 0.001$) and extent of disease was also important ($P = 0.01$). After adjustment for these two factors, treatment group had no significant effect on survival ($P = 0.30$). The 95% confidence interval for the ratio of hazard on the M group to that on the NoM group was (0.67, 1.13) and its estimate was 0.88. The median survival was 9 months in the M-group and 7.5 months in the NoM-group. These results agree with the results obtained by the Medical Research Council Lung Cancer Working Party (1989*b*). No justification for the trial size was included in the trial protocol or the published report.

### *Choice of a sequential design*

Among the sequential stopping rules appropriate for this trial, the *single* triangular design was chosen on the basis of its asymmetric power requirement and minimum sample size considerations (see Whitehead, 1993). Asymmetric designs require a high probability to detect superiority of the experimental treatment while the probability to detect its inferiority is relaxed. This seemed appropriate in this trial: it was important to produce conclusive evidence of superiority of additional maintenance chemotherapy (M) if it was indeed effective. However, if it was ineffective, it was less important to prove this. Given its toxicity, clinicians would only choose to use M if it were superior.

The triangular design would lead to stopping the study if either a striking advantage for the M group became apparent, or if no advantage emerged. The rapid result in the case of no advantage, allowing recruitment to M to be closed, was important given the toxicity of the chemotherapy. In the case of a moderate improvement in survival due to maintenance chemotherapy, the design guaranteed a larger sample size, which was then desirable to obtain a more reliable estimate of the treatment difference. This larger sample size was nonetheless very likely to be smaller than that of a conventional design.

The triangular test was designed to detect a difference between the treatments measured in terms of improvement in survival on M chemotherapy compared with NoM. The analysis performed by the MRC took place when 235 deaths had accumulated. The median survival in the NoM group was anticipated to be 8 months. To make the sequential study comparable, it was assumed that the aim of the trial was to give a 90% chance of detecting as significant at the 5% level (two-sided alternative) an increased 8-month survival to 0.64 in the M group. Such a treatment difference corresponds to a hazard ratio (M relative to NoM) of HR = 0.66.

Statistics Z and V described in the previous section were used. Assuming a recruitment rate of six patients per month, and interpolating the expected 32-week survival probabilities exponentially, it was anticipated that the required 235 deaths would accrue in 72 months. The design is shown in Figure 2a.

It can be seen from Table VI that the expected sample size under different magnitudes of treatment difference is well below 235, the number of deaths used in the conventional study. In the case of a modest improvement even the 90-th percentile of the number of deaths is not far above 235. The

**Table V** Analysis, estimation, size and duration at termination of the trial of chemotherapy and radiotherapy *vs* selective treatment in the management of small-cell lung cancer

| | *Median unbiased estimate of HR* | *95% C.I. for HR* | P-*value* | *Duration (months)* | *Number deaths* | *Number patients* |
|---|---|---|---|---|---|---|
| Sequential | 0.47 | (0.28,0.82) | 0.008 | 24 | 67 | 94 |
| Overrunning | 0.54 | (0.33,0.88) | 0.014 | 72 | 93 | 94 |
| MRC procedure | 0.44 | (0.30,0.64) | <0.001 | 72 | 147 | 151 |

maximum number of deaths was 363, although continuation to this value is of negligible probability. Anticipating approximately ten looks as in the previous trial, the first interim look was scheduled for December 1982, 18 months after the first recruitment and subsequent looks at 6-month intervals.

### *Results from the sequential design*

Points (V, Z) were plotted at each inspection to determine whether to stop the trial. At the first interim look, no prognostic factors were detected to have a significant relationship with survival. A survival analysis, stratified for the extent of disease pre-treatment and degree of response at time of randomisation was used. These factors were allowed for when allocating patients to treatment, and so all analyses are adjusted for them.

The trial ended at the third interim analysis. The sample path is shown in Figure 2b. The trial concluded with acceptance of the null hypothesis, suggesting that there is no evidence that survival can be prolonged by a policy of continuing chemotherapy beyond the initial induction treatment of six courses. The *P*-value at the time of stopping was $P = 0.44$, the 95% confidence interval for the hazard ratio was (0.77, 1.06) and the median unbiased estimate was 1.19. All of these values allow for the previous interim analyses. Following the method described in the previous example, late results gathered from a follow up to 72 months were incorporated in the analysis, and a comparison with the sample path which would have emerged had the trial not been stopped is made. The results are shown in Figures 2c and d, and in Table VII.

The conclusions of the trial at the time of stopping and after overrunning are both in agreement with the trend exhibited by the sample path when the trial was left to

**Figure 2** **a**, The triangular test with significance level = 0.05, power = 0.90 and HR = 0.655 used for the sequential simulation of the MRC trial comparing maintenance *vs* non-maintenance chemotherapy in the management of small-cell lung cancer. **b**, Final plot of the sequential simulation of the MRC comparison of maintenance *vs* non-maintenance chemotherapy in the management of small-cell lung cancer. **c**, Overrunning to 72 months of the sequential simulation of the MRC comparison of maintenance *vs* non-maintenance chemotherapy in the management of small-cell lung cancer. **d**, Sequential follow-up of the accumulated data of the MRC comparison of maintenance *vs* non-maintenance chemotherapy in the management of small-cell lung cancer.

**Table VI** Properties at termination of the triangular test for the trial of maintenance *vs* no-maintenance chemotherapy in small-cell lung cancer

| *Hazard ratio* | *Proportion surviving beyond 8 months (M group)* | *Proportion surviving beyond 8 months (NoM group)* | *Expected number of deaths* | *Median of number of deaths* | *90-th percentile of number of deaths* |
|---|---|---|---|---|---|
| 1.236 | 0.430 | 0.5 | 78 | 73 | 117 |
| 1.000 | 0.500 | 0.5 | 117 | 108 | 189 |
| 0.809 | 0.570 | 0.5 | 168 | 164 | 254 |
| 0.655 | 0.635 | 0.5 | 142 | 133 | 227 |

**Table VII** Analysis, estimation, size and duration at termination of the trial of maintenance *vs* no-maintenance chemotherapy in small-cell lung cancer

| | *Median unbiased estimate of HR* | *95% C.I. for HR* | P-*value* | *Duration (months)* | *Number deaths* | *Number patients* |
|---|---|---|---|---|---|---|
| Sequential | 1.19 | (0.77,1.82) | 0.44 | 27 | 92 | 180 |
| Overrunning | 1.01 | (0.69,1.43) | 0.97 | 72 | 172 | 180 |
| MRC procedure | 0.88 | (0.67,1.13) | 0.30 | 72 | 255 | 265 |

continue beyond the third look until 72 months. The *P*-value reported by the MRC procedure was larger than that of the sequential analysis. The estimate of the hazard ratio from the sequential analysis is broadly similar to that from the fixed sample study. The confidence interval is wider for the sequential analysis but clearly covering one. This situation was also sufficient for the purpose of this trial: there was no need to recruit more patients to look for a more accurate estimate of a small treatment effect. Table VII also shows the substantial reductions brought by the sequential design. Recruitment was stopped 27 months after entry of the first patient and an analysis was produced. A reduction in the duration of the trial by 45 months was achieved. The reduction in the number of patients and the actual number of deaths obtained after a follow-up period of 72 months was also considerable: 85 recruitments and 83 deaths.

### Discussion

The comparative reanalyses performed for each of the two trials considered here show that the qualitative conclusions of significance were in agreement with one another. The sequential designs were completed more quickly and with fewer patients, whereas the MRC procedures achieved smaller *P*-values. Estimates of hazard ratios from the sequential designs were broadly similar to those from the MRC procedures, especially when overrunning was allowed. Confidence intervals from the sequential analyses are in general wider than those from fixed-sample analyses. In our reanalyses, the confidence intervals were wider in the sequential analyses even when overrunning was allowed. This situation was acceptable since there was no need to look for a more accurate estimation of a clearly large or small treatment effect, and therefore it was unethical to continue recruitment for this purpose. Of course, these comments apply to only two illustrative trials. Their general validity however follows from mathematical results presented in Whitehead (1992).

Both the triangular and the double triangular tests illustrated here were chosen under the assumption that the single end-point, duration of survival, was of principal interest. If secondary end-points such as those indicating quality of life are also going to be important in the final analysis, then a sequential design might be chosen which ensures that a relatively large sample is observed unless a major treatment difference becomes apparent. One such design is the restricted procedure (see Whitehead, 1993). In the second trial presented in this paper, quality of life endpoints were of interest in addition to survival. The relative merits of different sequential designs would have needed careful consideration had the trial been planned according to the methods of this paper.

It has to be admitted that our reanalyses were idealised in that all events occurring before 18 months, say, were included in the interim analyses conducted at 18 months. In practice there would be a reporting lag. However, management procedures could be instituted to maximise the information available at the time of each interim analysis. Furthermore, in retrospect it was difficult to know what planning predictions would have been made about recruitment rate, and hindsight based on the real trial results influenced the predictions which we used. Note however, that such predictions effect only statements about the likely duration of the study. If the predictions are wrong, the study design and analysis remain valid and the power is achieved. It just might take longer (or shorter) to complete the trial than had been first thought.

Although any sequential procedure requires a commitment to accumulate a sample size larger than that of the fixed sample design, these trials both reached a conclusion before the available information has been used up. The conclusions of the trials at the time of stopping and after overrunning have been in agreement with the trend exhibited by the sample path when the trial has been followed beyond the final look up to the time of the fixed-sample analysis.

Formal stopping rules have been taken up by the pharmaceutical industry in Europe and by the National Cancer Institute in the United States. The methodology has so far been applied to many studies, some of them involving lung cancer, leukaemia, stroke and respiratory distress in infants. A sequential design has been adopted by the Medical Research Couneil Urological Working Party: Renal Carcinoma Subgroup for a phase III study comparing the effect of a biological (α-Interferon) *vs* a hormonal (medroxy-progesterone acetate) therapy in the management of renal carcinoma (MRC Urological Working Party, 1991).

This work has been completed under Grant NO. G9008019 from the British Medical Research Council. The authors would like to thank the MRC Lung Cancer Working Party, for making the data available and for their interest and cooperation in this project. Also we are grateful for helpful comments made on earlier drafts by Dr D.J. Girling, Dr. M.K.B. Parmar, Professor N.M. Bleehen and Professor R.L. Souhami.

### References

ALTMAN, D.G. (1991). *Practical Statistics for Medical Research.* Chapman and Hall: London.

ARMITAGE, P., MCPHERSON, C.K. & ROWE, B.C. (1969). Repeated significance tests on accumulating data. *J. R. Stat. Soc.*, **A132**, 235–244.

ARMITAGE, P. (1975). *Sequential Medical Trials*, (2nd Ed), Oxford: Blackwell.

BRUNIER, H. & WHITEHEAD, J. (1993). *PEST3.0. Operating Manual.* University of Reading.

COLLETT, D. (1994). *Modelling Survival Analysis in Medical Research.* Chapman and Hall: London (in press).

COX, D.R. (1972). Regression models and life-tables. *J. R. Stat. Soc.*, **B 34,** 187–202.

LAN, K.K.G. & DEMETS, D.L. (1983). Discrete sequential boundaries for clinical trials. *Biometrika*, **70,** 659–663.

MACHIN, D. (1992). Interim analysis and ethical issues in the conduct of trials. In Williams, C.J. (eds), pp. 203–215. *Introducing New Treatments for Cancer: Practical Ethical and Legal Problems.* John Wiley: Chichester.

MEDICAL RESEARCH COUNCIL LUNG CANCER WORKING PARTY (1989*a*). Survival, adverse reactions and quality of life during combination chemotherapy compared with selective palliative treatment for small-cell lung cancer. Report to the Medical Research Council by its Lung Cancer Working Party. *Respiratory Med.*, **83,** 51–58.

MEDICAL RESEARCH COUNCIL LUNG CANCER WORKING PARTY (1989*b*). Controlled trial of twelve versus six courses of chemotherapy in the treatment of small-cell lung cancer. Report to the Medical Research Council by its Lung Cancer Working Party. *Br. J. Cancer*, **59**, 584–590.

MEDICAL RESEARCH COUNCIL UROLOGICAL WORKING PARTY: RENAL CARCINOMA SUBGROUP (1991). *Metastatic Renal Carcinoma.* A randomised trial of α-Interferon v Medroxyprogesterone Acetate. Study Protocol RE01, MRC Cancer Trials Office, Cambridge.

O'BRIEN, P.C. & FLEMING, T.R. (1979). A multiple testing procedure for clinical trials. *Biometrics*, **35**, 549–556.

PEACE, K.E. (1992). *Biopharmaceutical Sequential Statistical Applications.* Marcel Dekker, Inc.: New York.

PETO, R., PIKE, M.C., ARMITAGE, P., BRESLOW, N.E., COX, D.R., HOWARD, S.V., MANTEL, N., MCPHERSON, K., PETO, J. & SMITH, P.G. (1976). Design and analysis of randomised clinical trials requiring prolonged observation of each patient. Introduction and design. *Br. J. Cancer*, **34**, 585–612.

POCOCK, S.J. (1977). Group sequential methods in the design and analysis of clinical trials. *Biometrika*, **64**, 191–199.

ROSNER, G.L. & TSIATIS, A.A. (1989). The impact that group sequential test would have made an ECOG clinical trials. *Statistics in Med.*, **8**, 505–516.

WHITEHEAD, J. (1992*a*). *The Design and Analysis of Sequential Clinical Trials*, (Second Edition). Chichester: Ellis Horwood.

WHITEHEAD, J. (1992*b*). Overrunning and underrunning in sequential clinical trials. *Controlled Clinical Trials*, **13**, 106–121.

WHITEHEAD, J. (1993). Interim analyses and stopping rules in cancer clinical trials. *Br. J. Cancer*, **68**, 1179–1185.